# A Perspective on Wastewater and Environmental Surveillance as a Public Health Tool for Low- and Middle-Income Countries

**DOI:** 10.3390/microorganisms13020238

**Published:** 2025-01-22

**Authors:** Mohammad Shehryaar Khan, Christian Wurzbacher, Anna Uchaikina, Boris Pleshkov, Olga Mirshina, Jörg E. Drewes

**Affiliations:** 1 Chair of Urban Water Systems Engineering, Technical University of Munich, Am Coulombwall 3, 85748 Garching, Germany; shehryaar.khan@tum.de (M.S.K.); anna.uchaikina@tum.de (A.U.); jdrewes@tum.de (J.E.D.); 2 Sanitary-Epidemiological Welfare and Public Health Committee of the Republic of Uzbekistan (SANEPIDCOM), Bunyodkor Street 46, Tashkent 100097, Uzbekistan

**Keywords:** wastewater-based epidemiology, wastewater and environmental surveillance (WES), antimicrobial resistance, food-, water-, and vector-borne illnesses, low- and middle-income countries

## Abstract

Geographical variations in infectious diseases create differences in public health priorities between high- and low-income countries. Low- and middle-income countries (LMICs) face resource constraints that limit adherence to international monitoring standards for wastewater-based epidemiology (WBE). The development of low-cost WBE programs, such as those to detect SARS-CoV-2, offers LMICs a promising tool for monitoring pathogens of local concern. In this work, we summarize important wastewater biomarkers for LMICs and their associated public health challenges, ranging from pathogens causing gastroenteritis to putative markers for plant diseases linked to food safety, as well as antimicrobial resistance. We raise awareness of the great potential of WBE for LMICs and highlight the critical health markers, research needs, and strategies necessary to establish tailored wastewater surveillance programs.

## 1. Introduction

The One Health program, established in 2006, has garnered traction for its focus on protecting human, animal, and environmental health through recommending policies to detect and prevent the spread of disease across the three groups. A joint panel, the One Health High-Level Expert Panel, was established in 2021 [1] to highlight the need for novel approaches to detect diseases early and share data among cooperating partners by “[s]urveillance, early detection and rapid data sharing in the prevention of emerging zoonoses” [2].

The importance of wastewater-based epidemiology (WBE) increased with the arrival of the coronavirus disease 19 (COVID-19) pandemic, and new epidemiological tools have been brought into focus. WBE has gained global recognition as an innovative tool for monitoring public health, particularly after its widespread use during the COVID-19 pandemic [3]. Recent advancements in WBE have expanded its scope to monitor pathogens, hazardous chemicals, and antimicrobial resistance (AMR) [4,5,6,7,8,9]. WBE, which was first introduced to detect the polio virus in 1940 by Dr. John Paul and Dr. James Trask [10], was later used for to track occurrences of cytopathic viruses in Finland in the 1960s and two decades later to monitor polio in Israel [11,12]. With COVID-19, governments around the globe became interested in adopting WBE, leading to a multitude of studies in data analysis and processing strategies of sample data (like active or passive sampling, RNA extraction protocols, and utilizing quantitative or digital PCR) [13,14,15,16,17,18]. This was followed by the European Union (EU) commission recommended establishing wastewater surveillance for severe acute respiratory syndrome 2 (SARS-CoV-2) and its variants of concern in 2021 [13].

Over the last years, a variety of technologies have been adopted to detect biomarkers in wastewater because of research into SARS-CoV-2 monitoring and Singh et al. provide a review of such methods [19]. Polymerase Chain Reaction (PCR) and quantitative PCR (qPCR) methods were initially used to detect (and quantify in the case of qPCR) SARS-CoV-2, but the difference in sensitivities of the assays, along with inhibition effects from the wastewater matrix, decrease the accuracy of the results [20]. Ho et al. developed a digital droplet PCR (ddPCR) protocol that enabled the target genes’ absolute quantification with negligible interference using duplex assays [20], and another study adapted digital PCR (dPCR) [18]. dPCR and ddPCR machinery have a very low detection limit compared to qPCR [20]. Moreover, multiplex kits, including pentaplex kits [21], have undergone rapid development, enabling the quantification of multiple genes in a single sample well [18,20,21]. Newer technologies, such as Next Generation Sequencing (NGS) and Loop-mediated isothermal amplification (LAMP), are being incorporated into the detection of pathogens and biomarkers [5,19,22,23]. While each detection method has its advantages and disadvantages, a variety of technologies can be integrated into a wastewater surveillance system.

On the other hand, the COVID-19 pandemic highlighted the inequality of the impact between high-income countries (HICs) and low- and middle-income countries (LMICs) in terms of the economic effect [24], food security [25], and overall disease burden [26]. Monitoring public health and diseases in LMICs is often fragmented, and statistics collected and utilized in high-income countries (HIC) are often unavailable and too resource-intensive for LMICs to gather [16]. Clinical testing carries a high economic cost and does not reveal an accurate trend of infection in the population [16]. Through mass surveillance via a sewer shed, an economic supplementary (or alternative) monitoring method is available for LMICs [16,27,28]. Among the many recommendations for tackling pathogenic threats, the WHO strongly advises developing a surveillance system for monitoring pathogens to protect animals, crops, and wild plants [29]. More importantly for LMICs, WBE offers a low-cost and non-invasive solution for health authorities to monitor the population without requiring individual testing, thereby reducing expenses [27,28]. WBE can serve as an early warning system for current and future pathogens of concern [16,19,27] and provides a more precise representation of the spread of a disease despite potentially missing data from individual testing (e.g., due to the lack of mandatory testing, inaccessibility to diagnostic resources, etc.) [27,28]. SARS-CoV-2 monitoring pipelines were established in many LMICs, and these systems can be further expanded upon for new targets [16,30]. In areas with less developed sanitation systems, surveilling surface water downstream from human conurbations has similarly been determined to be a feasible avenue for WBE [31,32]. However, while pandemics like COVID-19 are globally important, the specific needs of LMICs, which currently lack resources and attention, differ substantially from the HICs.

International institutions and experts can assist in tailoring WBE to LMICs for systematic and cost-efficient approaches; knowledge exchange and discussion forums for various technical and scientific hurdles regarding WBE can greatly assist LMICs. The EU Commission Joint Research Centre and the Health Emergency Preparedness and Response (HERA) initiated a global surveillance program supported through the EU at the internationally organized Global Consortium for Wastewater and Environmental Surveillance for Public Health (GLOWACON) [33], where representatives from all sectors share their approaches, findings, and innovations with the consortium. Furthermore, the EU Wastewater Observatory for Public Health allows the international community to share their monitoring activities through town hall meetings. It is currently facilitating the establishment of the EU wastewater academy, directing at developing specialized approaches for LMICs and HICs [34]. Considering the vulnerabilities and higher infectious disease burden in LMICs, international collaborations to refine WBE approaches should greatly assist LMICs in containing the spread of disease through public health monitoring tools.

HIC research, funding, and their WBE programs focus on pathogens like SARS-CoV-2, Influenza, or RSV [35,36,37,38,39] that are not as relevant for LMICs, where other diseases like gastrointestinal outbreaks are more important. The burden of infectious diseases, particularly food-, water-, and vector-borne illnesses, is much higher in LMICs than in HICs [40], and, therefore, would have increased relevance and attention than in HICs. For example, diarrhea is the second highest cause of mortality in children under 5 years old [41] and the seventh highest entirely for low-income countries in 2021 [42]. LMICs also face 249 million cases and 608,000 deaths from malaria (in 2022) and 1.3 to 4 million cases of cholera [43,44], with actual incidences of cholera being reported most in the Middle East, South Asia, and Africa [45]. The focus on WBE implementation in LMICs is still one step behind due to other challenges for establishing infrastructural and resource limitations and the need for an adaptive and economic approach are more pressing [40,46].

In this work, we highlight the different needs of LMICs in terms of WBE biomarkers of interest, which address interests of public health. We argue that in LMICs, the One Health concept is not only a political concept but a reality, as we have observed closed cycles between agriculture, food security, food-transmitted diseases, waste, and public health. Most practices, like using wastewater or sludge directly for agriculture, are no longer used in many HICs, and, therefore, the public health needs differ across countries. Addressing these intertwined challenges requires adaptive interventions and identifying the vulnerabilities and evaluating the resilience of LMICs.

## 2. Fecal–Oral Transmitted Diseases

Gastroenteritis is a digestive tract infection caused by fecal–oral transmission of external agents such as bacteria, viruses, and protozoa [43]. Table A1 presents a list of agents that are, in contrast to HIC, vital for LMICs. The disease commonly spreads through contaminated food, water, and surfaces, resulting in the patient suffering from diarrhea and dehydration [47]. Diarrheal diseases caused the third most Disability-Adjusted Life Years (DALYs) globally for all ages and fifth most for 0 to 9-year-old children, according to the Global Burden of Disease (GBD) 2019 report [48]. Young children are most vulnerable to diarrheal diseases, especially in countries with limited access to physicians or healthcare services [49,50].

In total, 453,000 deaths in children under five years old in 2008 were attributed to rotavirus-induced diarrhea [51]. Almost half of the deaths of children under five years of age from Rotavirus deaths in 2013 were in Nigeria, the Democratic Republic of Congo, Pakistan, and India [52]. Despite the global vaccination campaigns against Rotavirus, as of 2017, 67% of infants did not have access to the vaccine, and 80 countries globally have not incorporated the vaccine into national vaccination campaigns [53]. On a broader scale, noroviruses are the most common cause of gastroenteritis globally and affect all age groups, especially children and older adults [54,55]. Noroviruses are known to cause around 212,000 mortalities per year [56]. Although it is more prevalent in cases of gastroenteritis, detection and mitigation mechanisms for noroviruses are lacking in resources [50,53]. LMICs account for 82% of the disease burden and 97% of the deaths by norovirus, while the remainder is in high-income countries [57]. Studies found that norovirus costs $60.3 billion in societal and $4.2 billion in direct costs annually, with productivity losses between 84 and 99% across different regions [57]. While research and incorporation of norovirus and rotavirus detection are present in HICs, their current research focus is on other targets (e.g., monkeypox) [58,59,60,61,62,63].

Shigellosis was the second deadliest diarrheal disease between 1990 and 2016 globally, accounting for 13.2% of the deaths [64]. It is transmitted by the fecal–oral route, through flies, and raw vegetables irrigated with contaminated water [65,66]. Multiple studies on the detection of enteropathogens in wastewater and fecal samples also detected different *Shigella* serotypes [65,67,68,69,70,71,72]. Detecting *Shigella* specifically in wastewater samples or with EIEC (enteroinvasive *E. coli*) can be carried out by incorporating their quantification kits into a wastewater surveillance system. Furthermore, there are concerns regarding antibiotic resistance in *Shigella* species, which may have a stronger effect on LMIC residents due to overprescription of antibiotics, if viral or parasitic pathogens cause gastroenteritis and not *Shigella* [70,72,73,74,75].

All enteropathogens are excellent targets for WBE as they are transmitted via feces [70,76,77]. Valdivia-Carrera et al. (2023) determined a 4-week leading time for detecting high concentrations of Rotavirus in wastewater in the Peruvian Highlands before a gastroenteritis outbreak was reported. This further also suggests a lead time for LMIC governments to take preventive measures for mitigating the spread of gastroenteritis [78].

## 3. One Health Markers Relevant to Closed Cycles

Most scientific and diagnostic focus is on human health, while research into the WBE of animal and plant pathogens and their interlinkages is scarce. It is important to consider the interdependencies sketched by the One Health concept. The importance of zoonotic pathogens that spread through contact between animals and humans has been researched (e.g., H1N1 and SARS-CoV-2) [79], and LMICs with closed One Health cycles are moving into the center of emerging zoonotic diseases. However, other linkages that link plant, animal, and human health are lacking. Plants are the primary food for livestock and constitute over 80% of human food [80]. Plant disease is also a significant contributor to global food insecurity, with countries with agricultural-centered economies suffering substantial losses in trade. One example is the Coffee wilt disease caused by *Fusarium xylarioides*, which resulted in economic losses of up to USD 1 billion [81] and severely impacted countries dependent on coffee exports, such as Uganda [81,82], with a severe impact on income and too high costs to manage the spread of the disease [83]. Thus, these diseases lead to food insecurities and reduced capacity to mitigate the spread of plant diseases such as soybean, rice, maize, wheat, and potato [84]. The need to detect and surveil plant diseases can serve as an early detection system to help identify, control, and mitigate its spread [81]. WBE can serve as a stepping stone in advancing human and plant health monitoring and allow authorities to take proactive measures instead of reactionary ones. For example, one study detected two dozen plant pathogens in wastewater [85]. Experiments with wastewater discovered that Tobamoviruses retain infectivity even after wastewater treatment, presenting a challenge regarding water reuse for irrigation [86,87,88] and underlining the closed One Health cycles in LMICs. Multiple approaches should be considered for detecting plant pathogens [89,90,91,92]. A comprehensive list of publications on the detection of plant pathogens through different media is presented in Table A2.

## 4. Antimicrobial Resistance in One Health Cycles and Its Paradox for HICs

Similarly, antimicrobial resistance (AMR) continues to be a concern in One Health cycles, with AMR contributions from veterinary medicine through sewage or foodborne interactions [93]. Concern was raised over the strengthening and spread of AMR on a global scale due to inadequate regulation of antimicrobial pharmaceutical distribution [94,95,96,97,98]. While HICs focus on integrating AMR surveillance into WBE, LMICs require tailored approaches supported by international partnerships to address resource limitations and unique pathogen profiles [7,8,99,100,101]. Wastewater can act as an AMR reservoir and spread resistance into the environment through horizontal gene transfer, as studies have detected antimicrobial-resistant pathogens and genes in hospitals, nursing homes, and wastewater effluent [4,6,102,103]. Enteropathogens have been noted to have developed resistance to conventional and affordable medicine, and a consistent need to develop more potent antimicrobials carries a risk of continuing this cycle [95,96]. Pathogens commonly detected in LMICs, such as *S. Typhi* and non-*Typhi* species, *Vibrio cholerae*, *Mycobacterium tuberculosis*, *Shigella* spp., *Enterococcus* spp., *Candida auris*, and *E. Coli* have developed resistance and may become more difficult to contain and treat over time [6,66,75,96,103,104,105].

AMR monitoring has been at the center of global attention, with HICs investigating the prospects of its integration into wastewater-based epidemiology [103,106,107,108]. Klein et al. (2018) indicated that the increase of 65% in global antibiotic consumption between the years 2000–2015 was due to higher consumption in LMICs [109]. Economic and social factors, such as poverty and lack of access to authentic and high-quality medicine, expose the public in LMICs on a stronger magnitude compared to HICs. A lack of financial resources and standardized frameworks of monitoring, substandard drugs, and improper and unregulated use of antimicrobials contribute to strengthening AMR in LMICs [110,111]. This carries health, economic, and social implications for families with limited financial resources [95]. Balasubramanian and their team estimated 136 million hospital-associated drug-resistant infections (HARIs) per year, with the bulk of these infections being in Pakistan, India, and China [112]. This presents a dire need for regulating antibiotic distribution and consumption while monitoring for proactive measures. Calls to action have been made to monitor and reduce the prevalence of antimicrobial drugs in the environment to mitigate the spread of AMR [8,98]. Lack of regulation and control in LMICs can be a larger contributor to AMR originating in LMICs and eventually spreading into HICs [110]. Therefore, global collaboration for AMR monitoring has been encouraged and adopted, and the Global AMR Surveillance System (GLASS) can be combined with wastewater-based epidemiology to ease the burden of isolate testing and meet GLASS requirements for each hospital, clinic, or pharmacy and provide an overview of developing resistances [8,113]. Like the Stanford-led WastewaterSCAN project, AMR targets can be integrated into WBE operations [16,30,114,115,116]. Researchers in Niger used samples taken for the polio surveillance programs to monitor AMR over four years [117]. The potential of including AMR in existing LMIC WBE systems is promising, pending technical and scientific support from the institutes and scientific community from the HICs.

## 5. Change in Perspective and Research Needs with an LMIC-Centric View

The burden of diseases and AMR: Outbreaks of food-, water- and vector-borne illnesses continue to affect the public economically and socially, especially in countries with limited resources. To further exacerbate the issue, pathogens increasingly develop antimicrobial resistance to conventional and affordable treatment [4,95,96]. Pathogens that can be transmitted via water and vectors, such as mosquitoes, have also shown promise in being detected in wastewater samples. An overview of the detection of biomarkers, such as the Zika, Chikungunya, and Dengue viruses, *Plasmodium* spp., etc., is included in Table A3 for a detailed analysis.

WBE as a cost-effective solution for One Health monitoring: While biomarkers of interest vary on a regional or local scale, it is essential to investigate and expand on prevalent pathogens in countries with limited resources and a burdened health infrastructure. With the ever-growing strain on the LMIC health sectors and their vulnerabilities to an epidemic or pandemic, an effective and economical tool is required to monitor and mitigate the spread of diseases. WBE can be a cost-effective tool to detect and monitor biomarkers, enabling quicker responses in policymaking to protect public health and welfare.

Leveraging established surveillance systems: Existing wastewater surveillance systems for SARS-CoV-2, polio, and other targets can be configured to monitor infectious diseases. Studies have successfully detected biomarkers in wastewater through diverse approaches, and these can be adapted and incorporated into the system. WBE offers not only progress towards adapting the One Health approach but can assist in achieving Sustainable Development Goal 3 aimed at ending the spread of communicable diseases [118].

This effort, however, cannot be made by LMICs alone due to constrained resources and knowledge capacity. To bridge the gap in global health, HICs need to assist in developing and implementing the detection of biomarkers relevant to LMICs and, thereby, contain the spread whilst alleviating the public health burden.

## Data Availability

No new data were created for this research.

## Abbreviations

The following abbreviations are used in this manuscript:

AMR	Antimicrobial Resistance
COVID-19	Coronavirus Disease 2019
EU	European Union
HIC	High-Income Countries
LAMP	Loop-mediated isothermal amplification
LMIC	Low- and Middle-Income Countries
NGS	Next Generation Sequencing
PCR	Polymerase Chain Reaction
dPCR	Digital Polymerase Chain Reaction
ddPCR	Digital Droplet Polymerase Chain Reaction
qPCR	Quantitative Polymerase Chain Reaction
RT-PCR	Reverse Transcription Polymerase Chain Reaction
SARS-CoV-2	Severe Acute Respiratory Syndrome Coronavirus 2
WBE	Wastewater-Based Epidemiology
WES	Wastewater and Environmental Surveillance
WHO	World Health Organization

## Appendix A

**Table A1:** Publications detecting Rotaviruses, Noroviruses G1/G2, and Adenoviruses 40/41 in waste mediums through different methods.

Matrix	Rotavirus	Norovirus G1/G2	Adenovirus 40/41	Detection Method	Source
Influent	✓	✓	✓	qPCR	[60]
Influent and effluent	✓	✓	✓	Nested PCR	[119]
Influent	✓	✓	✓	ddPCR	[120]
Influent and effluent		✓	✓	qPCR	[59]
Influent and effluent		✓	✓	qPCR	[121]
Wastewater (screened influent and treated stream)	✓	✓		Semi-nested PCR	[122]
Influent		✓	✓	q- and RT-PCR	[5]
Secondary effluent		✓		qPCR	[123]
Influent and effluent		✓	✓	qPCR	[58]
Influent and effluent from four different treatment technologies		✓		qPCR	[124]
Domestic sewage, treated wastewater		✓		RT-PCR	[125]
Influent		✓		qPCR	[126]
Effluent			✓	qPCR (Type 41)	[127]
Influent			✓ ^c^	qPCR	[128]
Fecal, raw and treated wastewater, and sludge			✓ ^c^	qPCR	[129]
Beach water			✓	q- and RT-PCR	[130]
Fecal samples	✓	✓	✓	Multiplex PCR	[76]
Fecal samples	✓	✓	✓	RT-PCR	[131]
Fecal samples	✓	✓		RT-PCR	[132]
Fecal samples	✓	✓	✓	qPCR assay	[133]
Fecal samples		✓		RT-PCR	[134]
Fecal samples	✓	✓	✓	Enzyme-immunoassay, RT-PCR	[135]
Fecal samples	✓ ^a^		✓ ^a^	Immunochromatographic assay	[136]
Fecal samples	✓ ^a^	✓ ^b^	✓ ^a^	Enzyme immunoassay, PCR	[137]
Fecal samples	✓ ^a^	✓ ^a^	✓ ^a^	Enzyme-linked immunosorbent assay (ELISA)	[138]
Fecal samples	✓ ^a^		✓ ^a^	Immunochromatographic assay	[70]
Fecal samples	✓ ^a^		✓ ^a^	Immunochromatographic assay	[72]

^a^—the antigens for the virus were tested. ^b^—both antigens and the virus were tested. ^c^—primers detect all members of the human adenovirus group.

**Table A2:** Plant Pathogens and their detection in different matrices.

Disease	Biomarker	Matrix	Detection Method	Source
Mottling	Bell pepper mottle virus (BPeMV), cucumber green mottle mosaic virus (CGMMV), pepper mild mottle virus (PMMoV), tobacco mild green mosaic virus (TMGMV), tomato brown rugose fruit virus (ToBRFV), tomato mosaic virus (ToMV), tomato mottle mosaic virus (ToMMV), and tropical soda apple mosaic virus (TSAMV)	Wastewater	Sequencing	[139]
	Tobacco mosaic virus (TMV), Cucumber green mottle mosaic virus (CGMMV), Cucumber mosaic virus (CMV), Tomato mosaic virus (ToMV), Tomato mottle mosaic virus (ToMMV), Tomato chlorosis virus (ToCV), Pepper mild mottle virus (PMMoV)	Surface water	RT-PCR	[88]
	*Phytophthora ramorum*	Isolates	PCR	
	*Phymatotrichopsis omnivora*	Plant tissue and soil samples	qPCR	
	*Pratylenchus penetrans*	Artificially infested soil samples	qPCR	
	*Austropuccinia psidii*	Grounded leaves	PCR	
Rugose	Tomato brown rugose fruit virus (ToBRFV)	Wastewater Influent	Sequencing	[87]
	46 other plant viruses			
Fusariosis	*Fusarium solani* species complex 1 (*FSSC 1*)	Wastewater Influent	dPCR	[140]
	*Fusarium oxysporum spf. lycopersici* and *Fusarium verticillioides*	Isolates	dPCR	[91]
	*Fusarium oxysporum* spf. *niveum*	Isolates	PCR	[141]
	*Phytophthora* spp.	Isolates	Lab-on-a-chip PCR	[142]
	*Phytophthora colocasiae*	Leaf and tubers	PCR	[143]
Yellowing of leaves, fruit yield reduction	Tomato yellow leaf curl virus (TYLCV)	Soil sample	qPCR	[88]
Plum Pox Disease	Plum pox virus (PPV)	Leaves	RT-qPCR	[144]
Citrus Greening	*Candidatus Liberibacter asiaticus*	Leaf petiole	qPCR and ddPCR	[145]
Anthracnose (for gourd family members)	*Colletotrichum lagenarium*	Infected fruits	PCR	[146]

**Table A3:** Publications detecting other relevant biomarkers in different matrices.

Disease	Biomarker	Matrix	Detection Method	Source
Malaria	*Plasmodium faciparum*	Urine and saliva	Nested PCR	[147]
	*Plasmodium* spp.	Feces	Semi-nested RT-PCR	[148]
		Feces	qPCR	[149]
		Feces, blood, and urine	RT-PCR	[150]
		Saliva, feces, and blood	Nested qPCR	[151]
Zika Fever	Zika virus (ZIKV)	Wastewater influent	dPCR	[152]
		Wastewater influent	N/A	[153]
		Wastewater influent	qPCR	[154]
		Urine	RT-PCR	[155]
		Urine and saliva	qPCR	[156]
		Urine, saliva, and semen	RT-PCR	[157]
Dengue	Dengue virus (DENV)	Wastewater influent	qPCR	[158]
		Wastewater influent	ddPCR	[159]
		Wastewater influent	qPCR	[154]
		Urine and saliva	RT-PCR	[160]
		Urine	RT-PCR	[161]
		Urine and saliva	qPCR	[162]
		Urine	RT-PCR	[163]
Chikungunya	Chikungunya (CHIKV) virus	Wastewater Influent	qPCR	[158]
		Urine and saliva	qPCR	[164]
Cholera	*Vibrio cholerae*	Primary influent and secondary effluent	PCR	[165]
		Wastewater from open drains and pumping stations	RT-PCR	[166]
		Seeded wastewater	PCR	[167]
		Wastewater network	qPCR	[168]
Typhoid Fever	*Salmonella Typhi*	Wastewater network	RT-PCR	[168]
		Wastewater network	qPCR	[169]
		Seeded Influent	qPCR	[170]
Tuberculosis	*Mycobacterium* spp.	Influent and effluent	ddPCR	[171]
		Influent and effluent	ddPCR	[172]
		Wastewater	PCR and electrophoresis	[173]
		Feces	PCR	[174]
Candida Auris	*Candida auris*	Raw influent and treated effluent	qPCR	[103]
		Influent, hospital wastewater, and sewer sample	qPCR	[175]
		Influent	Cell culture and qPCR	[176]
		Influent	ddRT-PCR	[120]
Fusariosis	*Fusarium* spp.	Seeded water and drain swab	Nested PCR	[177]
		Human blood, skin isolates	PCR	[178]
Aspergillosis	*Aspergillus Flavus*	Air and surface swabs at a wastewater treatment plant	qPCR	[179]

## References

[1] World Health Organization (2023). One Health High-Level Expert Panel (OHHLEP). https://www.who.int/groups/one-health-high-level-expert-panel/ohhlep-term-1/meetings-and-working-groups.

[2] Mettenleiter T.C., Markotter W., Charron D.F., Adisasmito W.B., Almuhairi S., Behravesh C.B., Bilivogui P., Bukachi S.A., Casas N., Becerra N.C. (2023). The One Health High-Level Expert Panel (OHHLEP). One Health Outlook.

[3] Gawlik B., Tavazzi S., Mariani G., Skejo H., Sponar M., Higgins T., Medema G., Wintgens T. (2021). SARS-CoV-2 Surveillance Employing Sewage: Towards a Sentinel System.

[4] Fouz N., Pangesti K.N.A., Yasir M., Al-Malki A.L., Azhar E.I., Hill-Cawthorne G.A., El Ghany M.A. (2020). The Contribution of Wastewater to the Transmission of Antimicrobial Resistance in the Environment: Implications of Mass Gathering Settings. Trop. Med. Infect. Dis..

[5] McCall C., Wu H., Miyani B., Xagoraraki I. (2020). Identification of multiple potential viral diseases in a large urban center using wastewater surveillance. Water Res..

[6] Triggiano F., Calia C., Diella G., Montagna M.T., De Giglio O., Caggiano G. (2020). The Role of Urban Wastewater in the Environmental Transmission of Antimicrobial Resistance: The Current Situation in Italy (2010–2019). Microorganisms.

[7] Iskandar K., Molinier L., Hallit S., Sartelli M., Hardcastle T.C., Haque M., Lugova H., Dhingra S., Sharma P., Islam S. (2021). Surveillance of antimicrobial resistance in low- and middle-income countries: A scattered picture. Antimicrob. Resist. Infect. Control.

[8] Pruden A., Vikesland P.J., Davis B.C., de Roda Husman A.M. (2021). Seizing the moment: Now is the time for integrated global surveillance of antimicrobial resistance in wastewater environments. Curr. Opin. Microbiol..

[9] Kasprzyk-Hordern B., Sims N., Jagadeesan K., Proctor K., Wade M.J., Jones D.L. (2023). Wastewater-based epidemiology for comprehensive community health diagnostics in a national surveillance study: Mining biochemical markers in wastewater. J. Hazard. Mater..

[10] Trask J.D., Paul J.R. (1942). PERIODIC EXAMINATION OF SEWAGE FOR THE VIRUS OF POLIOMYELITIS. J. Exp. Med..

[11] Pöyry T., Stenvik M., Hovi T. (1988). Viruses in sewage waters during and after a poliomyelitis outbreak and subsequent nationwide oral poliovirus vaccination campaign in Finland. Appl. Environ. Microbiol..

[12] Slater P., Costin C., Yarrow A., Ben-Zvi T., Avni A., Epstein I., Orenstein W., Morag A., Handsher R., Green M. (1990). Poliomyelitis outbreak in Israel in 1988: A report with two commentaries. Lancet.

[13] European Commission, Directorate-General for Environment Commission Recommendation (EU) 2021/472 of 17 March 2021 on a Common Approach to Establish a Systematic Surveillance of SARS-CoV-2 and Its Variants in Wastewaters in the EU C(1925). EUR-Lex2021. https://eur-lex.europa.eu/legal-content/EN/TXT/?uri=CELEX%3A32021H0472.

[14] Picó Y., Barceló D. (2021). Identification of biomarkers in wastewater-based epidemiology: Main approaches and analytical methods. TrAC Trends Anal. Chem..

[15] Rossmann K., Clasen R., Münch M., Wurzbacher C., Tiehm A., Drewes J.E. (2021). SARS-CoV-2 Crisis Management With a Wastewater Early-Warning System in the Bavarian District of Berchtesgadener Land, Germany. Dtsch. Arztebl. Int..

[16] Shrestha S., Yoshinaga E., Chapagain S.K., Mohan G., Gasparatos A., Fukushi K. (2021). Wastewater-Based Epidemiology for Cost-Effective Mass Surveillance of COVID-19 in Low- and Middle-Income Countries: Challenges and Opportunities. Water.

[17] Vitale D., Morales Suárez-Varela M., Picó Y. (2021). Wastewater-based epidemiology, a tool to bridge biomarkers of exposure, contaminants, and human health. Curr. Opin. Environ. Sci. Health.

[18] Mitranescu A., Uchaikina A., Kau A.S., Stange C., Ho J., Tiehm A., Wurzbacher C., Drewes J.E. (2022). Wastewater-Based Epidemiology for SARS-CoV-2 Biomarkers: Evaluation of Normalization Methods in Small and Large Communities in Southern Germany. ACS EST Water.

[19] Singh S., Ahmed A.I., Almansoori S., Alameri S., Adlan A., Odivilas G., Chattaway M.A., Bin Salem S., Brudecki G., Elamin W. (2024). A narrative review of wastewater surveillance: Pathogens of concern, applications, detection methods, and challenges. Front. Public Health.

[20] Ho J., Stange C., Suhrborg R., Wurzbacher C., Drewes J.E., Tiehm A. (2022). SARS-CoV-2 wastewater surveillance in Germany: Long-term RT-digital droplet PCR monitoring, suitability of primer/probe combinations and biomarker stability. Water Res..

[21] Li M., Lv Y., Cui D., Xu Y., Lin M., Zhang X., Wang Y., Shen C., Xie J. (2023). Development and clinical validation of a one-step pentaplex real-time reverse transcription PCR assay for detection of hepatitis virus B, C, E, Treponema pallidum, and a human housekeeping gene. BMC Infect. Dis..

[22] Alvarez-Serna B.E., Ramírez-Chavarría R.G., Castillo-Villanueva E., Carrillo-Reyes J., Ramírez-Zamora R.M., Buitrón G., Alvarez-Icaza L. (2023). Label-free and portable field-effect sensor for monitoring RT-LAMP products to detect SARS-CoV-2 in wastewater. Talanta.

[23] Oh C., Xun G., Lane S.T., Petrov V.A., Zhao H., Nguyen T.H. (2024). Portable, single nucleotide polymorphism-specific duplex assay for virus surveillance in wastewater. Sci. Total Environ..

[24] Josephson A., Kilic T., Michler J.D. (2021). Socioeconomic impacts of COVID-19 in low-income countries. Nat. Hum. Behav..

[25] Bong C.L., Brasher C., Chikumba E., McDougall R., Mellin-Olsen J., Enright A. (2020). The COVID-19 Pandemic: Effects on Low- and Middle-Income Countries. Anesth. Analg..

[26] Sleeman K.E., de Brito M., Etkind S., Nkhoma K., Guo P., Higginson I.J., Gomes B., Harding R. (2019). The escalating global burden of serious health-related suffering: Projections to 2060 by world regions, age groups, and health conditions. Lancet Glob. Health.

[27] Farkas K., Hillary L.S., Malham S.K., McDonald J.E., Jones D.L. (2020). Wastewater and public health: The potential of wastewater surveillance for monitoring COVID-19. Curr. Opin. Environ. Sci. Health.

[28] Mao K., Zhang K., Du W., Ali W., Feng X., Zhang H. (2020). The potential of wastewater-based epidemiology as surveillance and early warning of infectious disease outbreaks. Curr. Opin. Environ. Sci. Health.

[29] Hayman D.T., Adisasmito W.B., Almuhairi S., Behravesh C.B., Bilivogui P., Bukachi S.A., Casas N., Becerra N.C., Charron D.F., Chaudhary A. (2023). Developing One Health surveillance systems. One Health.

[30] Schmidt C. (2020). Watcher in the wastewater. Nat. Biotechnol..

[31] Guerrero-Latorre L., Ballesteros I., Villacrés-Granda I., Granda M.G., Freire-Paspuel B., Ríos-Touma B. (2020). SARS-CoV-2 in river water: Implications in low sanitation countries. Sci. Total Environ..

[32] Fongaro G., Rogovski P., Savi B.P., Cadamuro R.D., Pereira J.V.F., Anna I.H.S., Rodrigues I.H., Souza D.S.M., Saravia E.G.T., Rodríguez-Lázaro D. (2022). SARS-CoV-2 in Human Sewage and River Water from a Remote and Vulnerable Area as a Surveillance Tool in Brazil. Food Environ. Virol..

[33] European Commission Launching GLOWACON: A Global Initiative for Wastewater Surveillance for Public Health health.ec.europa.eu.2024. https://health.ec.europa.eu/latest-updates/launching-glowacon-global-initiative-wastewater-surveillance-public-health-2024-03-21_en.

[34] European Commission EU Wastewater Observatory for Public Health Wastewater-Observatory.jrc.ec.europa.eu.2025. https://wastewater-observatory.jrc.ec.europa.eu/#/content/page-about.

[35] Agrawal S., Orschler L., Lackner S. (2021). Long-term monitoring of SARS-CoV-2 RNA in wastewater of the Frankfurt metropolitan area in Southern Germany. Sci. Rep..

[36] La Rosa G., Mancini P., Ferraro G.B., Veneri C., Iaconelli M., Bonadonna L., Lucentini L., Suffredini E. (2021). SARS-CoV-2 has been circulating in northern Italy since December 2019: Evidence from environmental monitoring. Sci. Total Environ..

[37] Boehm A.B., Hughes B., Duong D., Chan-Herur V., Buchman A., Wolfe M.K., White B.J. (2023). Wastewater concentrations of human influenza, metapneumovirus, parainfluenza, respiratory syncytial virus, rhinovirus, and seasonal coronavirus nucleic-acids during the COVID-19 pandemic: A surveillance study. Lancet Microbe.

[38] Länsivaara A., Lehto K.M., Hyder R., Luomala O., Lipponen A., Hokajärvi A.M., Heikinheimo A., Pitkänen T., Oikarinen S. (2024). Wastewater-Based Surveillance of Respiratory Syncytial Virus Epidemic at the National Level in Finland. ACS EST Water.

[39] Toribio-Avedillo D., Gómez-Gómez C., Sala-Comorera L., Galofré B., Muniesa M. (2024). Adapted methods for monitoring influenza virus and respiratory syncytial virus in sludge and wastewater. Sci. Total Environ..

[40] Parkins M.D., Lee B.E., Acosta N., Bautista M., Hubert C.R., Hrudey S.E., Frankowski K., Pang X.L. (2024). Wastewater-based surveillance as a tool for public health action: SARS-CoV-2 and beyond. Clin. Microbiol. Rev..

[41] World Health Organization (WHO) (2024). Diarrhoea: World Health Organization (WHO). https://www.who.int/health-topics/diarrhoea.

[42] World Health Organization (WHO) (2024). The Top 10 Causes of Death. https://www.who.int/news-room/fact-sheets/detail/the-top-10-causes-of-death.

[43] World Health Organization (WHO) (2023). Malaria: World Health Organization. https://www.who.int/news-room/fact-sheets/detail/malaria.

[44] World Health Organization (WHO) (2023). Cholera: World Health Organization. https://www.who.int/news-room/fact-sheets/detail/cholera#:~:text=Researchers%20have%20estimated%20that%20each,treated%20with%20oral%20rehydration%20solution.

[45] European Centre for Disease Prevention and Control (ECDC) (2024). Cholera Worldwide Overview Solna. https://www.ecdc.europa.eu/en/all-topics-z/cholera/surveillance-and-disease-data/cholera-monthly.

[46] Grassly N.C., Shaw A.G., Owusu M. (2024). Global wastewater surveillance for pathogens with pandemic potential: Opportunities and challenges. Lancet Microbe.

[47] Walker-Smith J. (1988). Gastroenteritis. Diseases of the Small Intestine in Childhood.

[48] Vos T., Lim S.S., Abbafati C., Abbas K.M., Abbasi M., Abbasifard M., Abbasi-Kangevari M., Abbastabar H., Abd-Allah F., Abdelalim A. (2020). Global burden of 369 diseases and injuries in 204 countries and territories, 1990–2019: A systematic analysis for the Global Burden of Disease Study 2019. Lancet.

[49] Elliott E.J. (2007). Acute gastroenteritis in children. BMJ.

[50] Lee B., Damon C.F., Platts-Mills J.A. (2020). Pediatric acute gastroenteritis associated with adenovirus 40/41 in low-income and middle-income countries. Curr. Opin. Infect. Dis..

[51] Tate J.E., Burton A.H., Boschi-Pinto C., Steele A.D., Duque J., Parashar U.D. (2012). 2008 estimate of worldwide rotavirus-associated mortality in children younger than 5 years before the introduction of universal rotavirus vaccination programmes: A systematic review and meta-analysis. Lancet Infect. Dis..

[52] Tate J.E., Burton A.H., Boschi-Pinto C., Parashar U.D. (2016). Global, Regional, and National Estimates of Rotavirus Mortality in Children <5 Years of Age, 2000–2013. Clin. Infect. Dis..

[53] International Vaccine Access Center (2017). Gap Analysis of Rotavirus Vaccine Impact Evaluations in Settings of Routine Use Johns Hopkins Bloomberg School of Public Health.

[54] Glass R.I., Parashar U.D., Estes M.K. (2009). Norovirus gastroenteritis. N. Engl. J. Med..

[55] Hall A.J., Lopman B.A., Payne D.C., Patel M.M., Gastañaduy P.A., Vinjé J., Parashar U.D. (2013). Norovirus Disease in the United States. Emerg. Infect. Dis..

[56] Lopman B.A., Steele D., Kirkwood C.D., Parashar U.D. (2016). The Vast and Varied Global Burden of Norovirus: Prospects for Prevention and Control. PLoS Med..

[57] Bartsch S.M., Lopman B.A., Ozawa S., Hall A.J., Lee B.Y. (2016). Global Economic Burden of Norovirus Gastroenteritis. PLoS ONE.

[58] La Rosa G., Pourshaban M., Iaconelli M., Muscillo M. (2010). Quantitative real-time PCR of enteric viruses in influent and effluent samples from wastewater treatment plants in Italy. Ann. Dell’istituto Super. Sanita.

[59] Grøndahl-Rosado R.C., Yarovitsyna E., Trettenes E., Myrmel M., Robertson L.J. (2014). A One Year Study on the Concentrations of Norovirus and Enteric Adenoviruses in Wastewater and A Surface Drinking Water Source in Norway. Food Environ. Virol..

[60] Hellmér M., Paxéus N., Magnius L.O., Enache L., Arnholm B., Johansson A.M., Bergström T., Norder H. (2014). Detection of Pathogenic Viruses in Sewage Provided Early Warnings of Hepatitis A Virus and Norovirus Outbreaks. Appl. Environ. Microbiol..

[61] Tiwari A., Adhikari S., Kaya D., Islam A., Malla B., Sherchan S.P., Al-Mustapha A.I., Kumar M., Aggarwal S., Bhattacharya P. (2023). Monkeypox outbreak: Wastewater and environmental surveillance perspective. Sci. Total Environ..

[62] Wolfe M.K., Yu A.T., Duong D., Rane M.S., Hughes B., Chan-Herur V., Donnelly M., Chai S., White B.J., Vugia D.J. (2023). Use of Wastewater for Mpox Outbreak Surveillance in California. N. Engl. J. Med..

[63] Boehm Alexandria B., Shelden B., Duong D., Banaei N., White Bradley J., Wolfe Marlene K. (2024). A retrospective longitudinal study of adenovirus group F, norovirus GI and GII, rotavirus, and enterovirus nucleic acids in wastewater solids at two wastewater treatment plants: Solid-liquid partitioning and relation to clinical testing data. mSphere.

[64] Khalil I.A., Troeger C., Blacker B.F., Rao P.C., Brown A., Atherly D.E., Brewer T.G., Engmann C.M., Houpt E.R., Kang G. (2018). Morbidity and mortality due to shigella and enterotoxigenic *Escherichia coli* diarrhoea: The Global Burden of Disease Study 1990–2016. Lancet Infect. Dis..

[65] Ojha S.C., Yean Yean C., Ismail A., Banga Singh K.-K. (2013). A Pentaplex PCR Assay for the Detection and Differentiation of *Shigella* Species. BioMed Res. Int..

[66] Lampel K.A., Formal† S.B., Maurelli A.T. (2018). A Brief History of *Shigella*. EcoSal Plus.

[67] Thiem V.D., Sethabutr O., Von Seidlein L., Van Tung T., Canh D.G., Chien B.T., Tho L.H., Lee H., Houng H.S., Hale T.L. (2004). Detection of *Shigella* by a PCR assay targeting the ipaH gene suggests increased prevalence of shigellosis in Nha Trang, Vietnam. J. Clin. Microbiol..

[68] Pant A., Mittal A.K. (2008). New Protocol for the Enumeration of *Salmonella* and *Shigella* from Wastewater. J. Environ. Eng..

[69] Maheux A.F., Picard F.J., Boissinot M., Bissonnette L., Paradis S., Bergeron M.G. (2009). Analytical comparison of nine PCR primer sets designed to detect the presence of *Escherichia coli*/*Shigella* in water samples. Water Res..

[70] Bonkoungou I.J.O., Haukka K., Österblad M., Hakanen A.J., Traoré A.S., Barro N., Siitonen A. (2013). Bacterial and viral etiology of childhood diarrhea in Ouagadougou, Burkina Faso. BMC Pediatr..

[71] Lindsay B., Ochieng J.B., Ikumapayi U.N., Toure A., Ahmed D., Li S., Panchalingam S., Levine M.M., Kotloff K., Rasko D.A. (2013). Quantitative PCR for Detection of *Shigella* Improves Ascertainment of *Shigella* Burden in Children with Moderate-to-Severe Diarrhea in Low-Income Countries. J. Clin. Microbiol..

[72] Sire J.-M., Garin B., Chartier L., Fall N.K., Tall A., Seck A., Weill F.-X., Breurec S., Vray M. (2013). Community-acquired infectious diarrhoea in children under 5 years of age in Dakar, Senegal. Paediatr. Int. Child Health.

[73] Zaidi M.B., Estrada-García T. (2014). *Shigella*: A Highly Virulent and Elusive Pathogen. Curr. Trop. Med. Rep..

[74] Kotloff K.L., Riddle M.S., Platts-Mills J.A., Pavlinac P., Zaidi A.K.M. (2018). Shigellosis. Lancet.

[75] Puzari M., Sharma M., Chetia P. (2018). Emergence of antibiotic resistant *Shigella* species: A matter of concern. J. Infect. Public Health.

[76] Gaensbauer J.T., Lamb M., Calvimontes D.M., Asturias E.J., Kamidani S., Contreras-Roldan I.L., Dominguez S.R., Robinson C.C., Zacarias A., Berman S. (2019). Identification of Enteropathogens by Multiplex PCR among Rural and Urban Guatemalan Children with Acute Diarrhea. Am. J. Trop. Med. Hyg..

[77] Chacón L., Morales E., Valiente C., Reyes L., Barrantes K. (2021). Wastewater-Based Epidemiology of Enteric Viruses and Surveillance of Acute Gastrointestinal Illness Outbreaks in a Resource-Limited Region. Am. J. Trop. Med. Hyg..

[78] Valdivia-Carrera C.A., Ho-Palma A.C., Munguia-Mercado A., Gonzalez-Pizarro K., Ibacache-Quiroga C., Dinamarca A., Stehlík M., Rusiñol M., Girones R., Lopez-Urbina M.T. (2023). Surveillance of SARS-CoV-2, rotavirus, norovirus genogroup II, and human adenovirus in wastewater as an epidemiological tool to anticipate outbreaks of COVID-19 and acute gastroenteritis in a city without a wastewater treatment plant in the Peruvian Highlands. Sci. Total Environ..

[79] Xiao K., Zhang L. (2023). Wastewater pathogen surveillance based on One Health approach. Lancet Microbe.

[80] Rizzo D.M., Lichtveld M., Mazet J.A.K., Togami E., Miller S.A. (2021). Plant health and its effects on food safety and security in a One Health framework: Four case studies. One Health Outlook.

[81] Flood J. (2010). The importance of plant health to food security. Food Secur..

[82] Flood J. (2022). Coffee Wilt Disease.

[83] Peck L. (2023). Coffee Wilt Disease.

[84] Savary S., Willocquet L., Pethybridge S.J., Esker P., McRoberts N., Nelson A. (2019). The global burden of pathogens and pests on major food crops. Nat. Ecol. Evol..

[85] Zhang T., Breitbart M., Lee W.H., Run J.-Q., Wei C.L., Soh S.W.L., Hibberd M.L., Liu E.T., Rohwer F., Ruan Y. (2005). RNA Viral Community in Human Feces: Prevalence of Plant Pathogenic Viruses. PLoS Biol..

[86] Mehle N., Ravnikar M. (2012). Plant viruses in aqueous environment–Survival, water mediated transmission and detection. Water Res..

[87] Bačnik K., Kutnjak D., Pecman A., Mehle N., Žnidarič M.T., Aguirre I.G., Ravnikar M. (2020). Viromics and infectivity analysis reveal the release of infective plant viruses from wastewater into the environment. Water Res..

[88] Zhang X., Sun X., Liu Y., Qiao N., Wang X., Zhao D., Shang K., Zhu X. (2023). Potential risk of plant viruses entering disease cycle in surface water in protected vegetable growing areas of Eastern China. PLoS ONE.

[89] Tomlinson J.A., Boonham N., Hughes K.J., Griffin R.L., Barker I. (2005). On-site DNA extraction and real-time PCR for detection of Phytophthora ramorum in the field. Appl. Environ. Microbiol..

[90] Arif M., Fletcher J., Marek S.M., Melcher U., Ochoa-Corona F.M. (2013). Development of a rapid, sensitive, and field-deployable razor ex BioDetection system and quantitative PCR assay for detection of Phymatotrichopsis omnivora using multiple gene targets. Appl. Environ. Microbiol..

[91] Koo C., Malapi-Wight M., Kim H.S., Cifci O.S., Vaughn-Diaz V.L., Ma B., Kim S., Abdel-Raziq H., Ong K., Jo Y.-K. (2013). Development of a Real-Time Microchip PCR System for Portable Plant Disease Diagnosis. PLoS ONE.

[92] Baldi P., La Porta N. (2020). Molecular Approaches for Low-Cost Point-of-Care Pathogen Detection in Agriculture and Forestry. Front. Plant Sci..

[93] McEwen S.A., Collignon P.J. (2018). Antimicrobial Resistance: A One Health Perspective. Antimicrobial Resistance in Bacteria from Livestock and Companion Animals.

[94] Hart C., Kariuki S. (1998). Antimicrobial resistance in developing countries. BMJ.

[95] Byarugaba D.K. (2004). Antimicrobial resistance in developing countries and responsible risk factors. Int. J. Antimicrob. Agents.

[96] Okeke I.N., Laxminarayan R., Bhutta Z.A., Duse A.G., Jenkins P., O’Brien T.F., Pablos-Mendez A., Klugman K.P. (2005). Antimicrobial resistance in developing countries. Part I: Recent trends and current status. Lancet Infect. Dis..

[97] Holmes A.H., Moore L.S.P., Sundsfjord A., Steinbakk M., Regmi S., Karkey A., Guerin P.J., Piddock L.J.V. (2016). Understanding the mechanisms and drivers of antimicrobial resistance. Lancet.

[98] O’Neill J. (2016). Tackling Drug-Resistant Infections Globally: Final Report and Recommendations.

[99] Novo A., André S., Viana P., Nunes O.C., Manaia C.M. (2013). Antibiotic resistance, antimicrobial residues and bacterial community composition in urban wastewater. Water Res..

[100] Bäumlisberger M., Youssar L., Schilhabel M.B., Jonas D. (2015). Influence of a Non-Hospital Medical Care Facility on Antimicrobial Resistance in Wastewater. PLoS ONE.

[101] Pandey S., Pandey A.R. (2023). Strategy to Combat Antibiotic Resistance Bacteria and Genes in Wastewater in Developing Countries. https://www.preprints.org/manuscript/202301.0386/v1.

[102] Chau K., Barker L., Budgell E., Vihta K., Sims N., Kasprzyk-Hordern B., Harriss E., Crook D., Read D., Walker A. (2022). Systematic review of wastewater surveillance of antimicrobial resistance in human populations. Environ. Int..

[103] Barber C., Crank K., Papp K., Innes G.K., Schmitz B.W., Chavez J., Rossi A., Gerrity D. (2023). Community-Scale Wastewater Surveillance of *Candida auris* during an Ongoing Outbreak in Southern Nevada. Environ. Sci. Technol..

[104] Hale T.L., Keusch G.T. (1996). Shigella. Medical Microbiology.

[105] World Health Organization (WHO) (2022). Immunization, Vaccines and Biologicals WHO.int: WHO. https://www.who.int/teams/immunization-vaccines-and-biologicals/diseases/shigella.

[106] Łuczkiewicz A., Jankowska K., Fudala-Książek S., Olańczuk-Neyman K. (2010). Antimicrobial resistance of fecal indicators in municipal wastewater treatment plant. Water Res..

[107] Abdukhalilova G., Kaftyreva L., Wagenaar J.A., Tangyarikov B., Bektimirov A., Akhmedov I., Khodjaev Z., Kruse H. (2016). Occurrence and antimicrobial resistance of *Salmonella* and Campylobacter in humans and broiler chicken in Uzbekistan. Public Health Panor..

[108] Baba H., Nishiyama M., Watanabe T., Kanamori H. (2022). Review of Antimicrobial Resistance in Wastewater in Japan: Current Challenges and Future Perspectives. Antibiotics.

[109] Klein E.Y., Van Boeckel T.P., Martinez E.M., Pant S., Gandra S., Levin S.A., Goossens H., Laxminarayan R. (2018). Global increase and geographic convergence in antibiotic consumption between 2000 and 2015. Proc. Natl. Acad. Sci. USA.

[110] Sharma A., Singh A., Dar M.A., Kaur R.J., Charan J., Iskandar K., Haque M., Murti K., Ravichandiran V., Dhingra S. (2022). Menace of antimicrobial resistance in LMICs: Current surveillance practices and control measures to tackle hostility. J. Infect. Public Health.

[111] Rony M.K.K., Sharmi P.D., Alamgir H.M. (2023). Addressing antimicrobial resistance in low and middle-income countries: Overcoming challenges and implementing effective strategies. Environ. Sci. Pollut. Res..

[112] Balasubramanian R., Van Boeckel T.P., Carmeli Y., Cosgrove S., Laxminarayan R. (2023). Global incidence in hospital-associated infections resistant to antibiotics: An analysis of point prevalence surveys from 99 countries. PLoS Med..

[113] Sulis G., Sayood S., Gandra S. (2022). Antimicrobial resistance in low- and middle-income countries: Current status and future directions. Expert Rev. Anti-Infect. Ther..

[114] Gupta S., Venkatesh A., Ray S., Srivastava S. (2014). Challenges and prospects for biomarker research: A current perspective from the developing world. Biochim. Biophys. Acta (BBA)-Proteins Proteom..

[115] Stanford-Led WastewaterSCAN Project Adds Six New Disease Targets [Press Release]. StanfordReport2023. https://news.stanford.edu/stories/2023/10/stanford-led-wastewaterscan-project-adds-six-new-disease-targets.

[116] Naughton C.C., Roman F.A., Alvarado A.G.F., Tariqi A.Q., Deeming M.A., Kadonsky K.F., Bibby K., Bivins A., Medema G., Ahmed W. (2023). Show us the data: Global COVID-19 wastewater monitoring efforts, equity, and gaps. FEMS Microbes.

[117] Ousmane S., Kollo I.A., Jambou R., Boubacar R., Arzika A.M., Maliki R., Amza A., Liu Z., Lebas E., Colby E. (2023). Wastewater-Based Surveillance of Antimicrobial Resistance in Niger: An Exploratory Study. Am. J. Trop. Med. Hyg..

[118] World Health Organization (WHO) SDG Target 3.3|Communicable Diseases: By 2030, End the Epidemics of AIDS, Tuberculosis, Malaria and Neglected Tropical Diseases and Combat Hepatitis, Water-Borne Diseases and Other Communicable Diseases 2024. https://www.who.int/data/gho/data/themes/topics/indicator-groups/indicator-group-details/GHO/sdg-target-3.3-communicable-diseases#:~:text=Indicator%20Groups-,SDG%20Target%203.3%20%7C%20Communicable%20diseases%3A%20By%202030%2C%20end%20the,diseases%20and%20other%20communicable%20diseases.

[119] Symonds E.M., Griffin D.W., Breitbart M. (2009). Eukaryotic Viruses in Wastewater Samples from the United States. Appl. Environ. Microbiol..

[120] Alexandria B.B., Marlene K.W., Bradley J.W., Bridgette H., Dorothea D. (2023). Two years of longitudinal measurements of human adenovirus group F, norovirus GI and GII, rotavirus, enterovirus, enterovirus D68, hepatitis A virus, *Candida auris*, and West Nile virus nucleic-acids in wastewater solids: A retrospective study at two wastewater treatment plants. medRxiv.

[121] Masclaux F.G., Hotz P., Friedli D., Savova-Bianchi D., Oppliger A. (2013). High occurrence of hepatitis E virus in samples from wastewater treatment plants in Switzerland and comparison with other enteric viruses. Water Res..

[122] Ji Z., Wang X.C., Xu L., Zhang C., Rong C., Rachmadi A.T., Amarasiri M., Okabe S., Funamizu N., Sano D. (2019). Fecal Source Tracking in A Wastewater Treatment and Reclamation System Using Multiple Waterborne Gastroenteritis Viruses. Pathogens.

[123] Montazeri N., Goettert D., Achberger E.C., Johnson C.N., Prinyawiwatkul W., Janes M.E. (2015). Pathogenic enteric viruses and microbial indicators during secondary treatment of municipal wastewater. Appl. Environ. Microbiol..

[124] Silva AKd Saux J.-C.L., Parnaudeau S., Pommepuy M., Elimelech M., Guyader F.S.L. (2007). Evaluation of Removal of Noroviruses during Wastewater Treatment, Using Real-Time Reverse Transcription-PCR: Different Behaviors of Genogroups I and II. Appl. Environ. Microbiol..

[125] Ueki Y., Sano D., Watanabe T., Akiyama K., Omura T. (2005). Norovirus pathway in water environment estimated by genetic analysis of strains from patients of gastroenteritis, sewage, treated wastewater, river water and oysters. Water Res..

[126] Kazama S., Masago Y., Tohma K., Souma N., Imagawa T., Suzuki A., Liu X., Saito M., Oshitani H., Omura T. (2016). Temporal dynamics of norovirus determined through monitoring of municipal wastewater by pyrosequencing and virological surveillance of gastroenteritis cases. Water Res..

[127] Osuolale O., Okoh A. (2015). Incidence of human adenoviruses and Hepatitis A virus in the final effluent of selected wastewater treatment plants in Eastern Cape Province, South Africa. Virol. J..

[128] Sidhu J.P.S., Ahmed W., Toze S. (2013). Sensitive detection of human adenovirus from small volume of primary wastewater samples by quantitative PCR. J. Virol. Methods.

[129] Elmahdy E.M., Ahmed N.I., Shaheen M.N.F., Mohamed E.-C.B., Loutfy S.A. (2019). Molecular detection of human adenovirus in urban wastewater in Egypt and among children suffering from acute gastroenteritis. J. Water Health.

[130] Xagoraraki I., Kuo D.H.-W., Wong K., Wong M., Rose J.B. (2007). Occurrence of Human Adenoviruses at Two Recreational Beaches of the Great Lakes. Appl. Environ. Microbiol..

[131] Hall A.J., Rosenthal M., Gregoricus N., Greene S.A., Ferguson J., Henao O.L., Vinjé J., Lopman B.A., Parashar U.D., Widdowson M.A. (2011). Incidence of acute gastroenteritis and role of norovirus, Georgia, USA, 2004–2005. Emerg. Infect. Dis..

[132] Payne D.C., Vinje J., Szilagyi P.G., Edwards K.M., Staat M.A., Weinberg G.A., Hall C.B., Chappell J., Bernstein D.I., Curns A.T. (2013). Norovirus and medically attended gastroenteritis in U.S. children. N. Engl. J. Med..

[133] Liu J., Platts-Mills J.A., Juma J., Kabir F., Nkeze J., Okoi C., Operario D.J., Uddin J., Ahmed S., Alonso P.L. (2016). Use of quantitative molecular diagnostic methods to identify causes of diarrhoea in children: A reanalysis of the GEMS case-control study. Lancet.

[134] Giri S., Chhabra P., Kulkarni R., Reju S., Sabapathy S.K., Selvarajan S., Varghese T., Kalaivanan M., Dorairaj P., Kalrao V. (2024). Hospital-based norovirus surveillance in children <5 years of age from 2017 to 2019 in India. J. Med. Virol..

[135] Chhabra P., Payne D.C., Szilagyi P.G., Edwards K.M., Staat M.A., Shirley S.H., Wikswo M., Nix W.A., Lu X., Parashar U.D. (2013). Etiology of Viral Gastroenteritis in Children <5 Years of Age in the United States, 2008–2009. J. Infect. Dis..

[136] Celik C., Gozel M.G., Turkay H., Bakici M.Z., Güven A.S., Elaldi N. (2015). Rotavirus and adenovirus gastroenteritis: Time series analysis. Pediatr. Int..

[137] Wiegering V., Kaiser J., Tappe D., Weissbrich B., Morbach H., Girschick H.J. (2011). Gastroenteritis in childhood: A retrospective study of 650 hospitalized pediatric patients. Int. J. Infect. Dis..

[138] Ozdemir S., Delialioğlu N., Emekdaş G. (2010). Investigation of rotavirus, adenovirus and astrovirus frequencies in children with acute gastroenteritis and evaluation of epidemiological features. Mikrobiyoloji Bul..

[139] Rothman J.A., Whiteson K.L. (2022). Sequencing and Variant Detection of Eight Abundant Plant-Infecting Tobamoviruses across Southern California Wastewater. Microbiol. Spectr..

[140] Mehl H.L., Epstein L. (2008). Sewage and community shower drains are environmental reservoirs of *Fusarium solani* species complex group 1, a human and plant pathogen. Environ. Microbiol..

[141] Zhang Z., Zhang J., Wang Y., Zheng X. (2005). Molecular detection of *Fusarium oxysporum* f. sp. niveum and Mycosphaerella melonis in infected plant tissues and soil. FEMS Microbiol. Lett..

[142] Julich S., Riedel M., Kielpinski M., Urban M., Kretschmer R., Wagner S., Fritzsche W., Henkel T., Möller R., Werres S. (2011). Development of a lab-on-a-chip device for diagnosis of plant pathogens. Biosens. Bioelectron..

[143] Mishra A.K., Jeeva M.L., Vidyadharan P., Misra R.S., Hegde V. (2010). Rapid and sensitive detection of Phytophthora colocasiae associated with leaf blight of taro by species-specific polymerase chain reaction assay. Ann. Microbiol..

[144] Fotiou I.S., Pappi P.G., Efthimiou K.E., Katis N.I., Maliogka V.I. (2019). Development of one-tube real-time RT-qPCR for the universal detection and quantification of Plum pox virus (PPV). J. Virol. Methods.

[145] Selvaraj V., Maheshwari Y., Hajeri S., Chen J., McCollum T.G., Yokomi R. (2018). Development of a duplex droplet digital PCR assay for absolute quantitative detection of “Candidatus Liberibacter asiaticus”. PLoS ONE.

[146] Kuan C.-P., Wu M.-T., Huang H.C., Chang H. (2011). Rapid Detection of Colletotrichum lagenarium, Causal Agent of Anthracnose of Cucurbitaceous Crops, by PCR and Real-time PCR. J. Phytopathol..

[147] Mharakurwa S., Simoloka C., Thuma P.E., Shiff C.J., Sullivan D.J. (2006). PCR detection of *Plasmodium* falciparum in human urine and saliva samples. Malar. J..

[148] Jirků M., Pomajbíková K., Petrželková K.J., Hůzová Z., Modrý D., Lukeš J. (2012). Detection of *Plasmodium* spp. in human feces. Emerg. Infect. Dis..

[149] Keita A.K., Fenollar F., Socolovschi C., Ratmanov P., Bassene H., Sokhna C., Tall A., Mediannikov O., Raoult D. (2015). The detection of vector-borne-disease-related DNA in human stool paves the way to large epidemiological studies. Eur. J. Epidemiol..

[150] Al-Shehri H., Power B.J., Archer J., Cousins A., Atuhaire A., Adriko M., Arinaitwe M., Alanazi A.D., LaCourse E.J., Kabatereine N.B. (2019). Non-invasive surveillance of *Plasmodium* infection by real-time PCR analysis of ethanol preserved faeces from Ugandan school children with intestinal schistosomiasis. Malar. J..

[151] Imboumy-Limoukou R.K., Biteghe-Bi-Essone J.C., Lendongo Wombo J.B., Lekana-Douki S.E., Rougeron V., Ontoua S.S., Oyegue-Liabagui L.S., Mbani Mpega Ntigui C.N., Kouna L.C., Lekana-Douki J.B. (2023). Detection of *Plasmodium* falciparum in Saliva and Stool Samples from Children Living in Franceville, a Highly Endemic Region of Gabon. Diagnostics.

[152] Zhu K., Hill C., Muirhead A., Basu M., Brown J., Brinton M.A., Hayat M.J., Venegas-Vargas C., Reis M.G., Casanovas-Massana A. (2023). Zika virus RNA persistence and recovery in water and wastewater: An approach for Zika virus surveillance in resource-constrained settings. Water Res..

[153] Wong J.C.C., Tay M., Hapuarachchi H.C., Lee B., Yeo G., Maliki D., Lee W., Suhaimi N.-A.M., Chio K., Tan W.C.H. (2024). Zika Surveillance Complemented with Wastewater and Mosquito Testing. eBioMedicine.

[154] Chandra F., Lee W.L., Armas F., Leifels M., Gu X., Chen H., Wuertz S., Alm E.J., Thompson J. (2021). Persistence of Dengue (Serotypes 2 and 3), Zika, Yellow Fever, and Murine Hepatitis Virus RNA in Untreated Wastewater. Environ. Sci. Technol. Lett..

[155] Zhang F.-C., Li X.-F., Deng Y.-Q., Tong Y.-G., Qin C.-F. (2016). Excretion of infectious Zika virus in urine. Lancet Infect. Dis..

[156] Bonaldo M.C., Ribeiro I.P., Lima N.S., dos Santos A.A.C., Menezes L.S.R., da Cruz S.O.D., de Mello I.S., Furtado N.D., de Moura E.E., Damasceno L. (2016). Isolation of Infective Zika Virus from Urine and Saliva of Patients in Brazil. PLoS Neglected Trop. Dis..

[157] Bingham A.M., Cone M., Mock V., Heberlein-Larson L., Stanek D., Blackmore C., Likos A. (2016). Comparison of Test Results for Zika Virus RNA in Urine, Serum, and Saliva Specimens from Persons with Travel-Associated Zika Virus Disease-Florida, 2016. MMWR Morb. Mortal. Wkly. Rep..

[158] Monteiro S., Pimenta R., Nunes F., Cunha M.V., Santos R. (2023). Wastewater-based surveillance for tracing the circulation of Dengue and Chikungunya viruses. medRxiv.

[159] Wolfe M.K., Paulos A.H., Zulli A., Duong D., Shelden B., White B.J., Boehm A.B. (2024). Wastewater Detection of Emerging Arbovirus Infections: Case Study of Dengue in the United States. Environ. Sci. Technol. Lett..

[160] Poloni T.R., Oliveira A.S., Alfonso H.L., Galvão L.R., Amarilla A.A., Poloni D.F., Figueiredo L.T., Aquino V.H. (2010). Detection of dengue virus in saliva and urine by real time RT-PCR. Virol. J..

[161] Hirayama T., Mizuno Y., Takeshita N., Kotaki A., Tajima S., Omatsu T., Sano K., Kurane I., Takasaki T. (2012). Detection of Dengue Virus Genome in Urine by Real-Time Reverse Transcriptase PCR: A Laboratory Diagnostic Method Useful after Disappearance of the Genome in Serum. J. Clin. Microbiol..

[162] Andries A.-C., Duong V., Ly S., Cappelle J., Kim K.S., Try P.L., Ros S., Ong S., Huy R., Horwood P. (2015). Value of Routine Dengue Diagnostic Tests in Urine and Saliva Specimens. PLoS Neglected Trop. Dis..

[163] Van den Bossche D., Cnops L., Van Esbroeck M. (2015). Recovery of dengue virus from urine samples by real-time RT-PCR. Eur. J. Clin. Microbiol. Infect. Dis..

[164] Salles T.S., Sá-Guimarães T.E., Guimarães-Ribeiro V., Melo A.C.A., Ferreira D.F., Moreira M.F. (2021). Detection of Chikungunya Virus in Saliva and Urine Samples of Patients from Rio de Janeiro, Brazil. A Minim. Invasive Tool Surveill..

[165] Hounmanou Y.M.G., Mdegela R.H., Dougnon T.V., Mhongole O.J., Mayila E.S., Malakalinga J., Makingi G., Dalsgaard A. (2016). Toxigenic Vibrio cholerae O1 in vegetables and fish raised in wastewater irrigated fields and stabilization ponds during a non-cholera outbreak period in Morogoro, Tanzania: An environmental health study. BMC Res. Notes.

[166] Zohra T., Ikram A., Salman M., Amir A., Saeed A., Ashraf Z., Ahad A. (2021). Wastewater based environmental surveillance of toxigenic Vibrio cholerae in Pakistan. PLoS ONE.

[167] Barzamini B., Moghbeli M., Soleimani N.A. (2014). Vibrio cholerae Detection in Water and Wastewater by Polymerase Chain Reaction Assay. Int. J. Enteric Pathog..

[168] Chigwechokha P., Nyirenda R.L., Dalitsani D., Namaumbo R.L., Kazembe Y., Smith T., Holm R.H. (2024). Vibrio cholerae and *Salmonella* Typhi culture-based wastewater or non-sewered sanitation surveillance in a resource-limited region. J. Expo. Sci. Environ. Epidemiol..

[169] Uzzell C.B., Troman C.M., Rigby J., Mohan V.R., John J., Abraham D., Srinivasan R., Nair S., Meschke J.S., Elviss N. (2021). Environmental surveillance for *Salmonella* Typhi as a tool to estimate the incidence of typhoid fever in low-income populations. medRxiv.

[170] Zhou N., Ong A., Fagnant-Sperati C., Harrison J., Kossik A., Beck N., Shirai J., Burnor E., Swanstrom R., Demeke B. (2022). Evaluation of sampling and concentration methods for *Salmonella enterica* serovar Typhi detection from wastewater. medRxiv.

[171] Reddy P., Mtetwa H.N., Amoah I.D., Kumari S., Bux F. Wastewater-based epidemiology: An additional tool for TB surveillance?. Proceedings of the ISEE 2022: 34th Annual Conference of the International Society of Environmental Epidemiology.

[172] Mtetwa H.N., Amoah I.D., Kumari S., Bux F., Reddy P. (2023). Exploring the role of wastewater-based epidemiology in understanding tuberculosis burdens in Africa. Environ. Res..

[173] Suliman K., Siddique R., Nabi G., Sajjad W., Heenatigala P., Jingjing Y., Li Q., Hou H., Ali I. (2017). Investigation of Sewage and Drinking Water in Major Healthcare Centres for Bacterial and Viral Pathogens. Hydrol. Curr. Res..

[174] El Khéchine A., Henry M., Raoult D., Drancourt M. (2009). Detection of Mycobacterium tuberculosis complex organisms in the stools of patients with pulmonary tuberculosis. Microbiology.

[175] Babler K., Sharkey M., Arenas S., Amirali A., Beaver C., Comerford S., Goodman K., Grills G., Holung M., Kobetz E. (2023). Detection of the clinically persistent, pathogenic yeast spp. Candida auris from hospital and municipal wastewater in Miami-Dade County, Florida. Sci. Total Environ..

[176] Rossi A., Chavez J., Iverson T., Hergert J., Oakeson K., LaCross N., Njoku C., Gorzalski A., Gerrity D. (2023). *Candida auris* Discovery through Community Wastewater Surveillance during Healthcare Outbreak, Nevada, USA, 2022. Emerg. Infect. Dis..

[177] Graça M.G., van der Heijden I.M., Perdigão L., Taira C., Costa S.F., Levin A.S. (2016). Evaluation of two methods for direct detection of *Fusarium* spp. in water. J. Microbiol. Methods.

[178] Muraosa Y., Oguchi M., Yahiro M., Watanabe A., Yaguchi T., Kamei K. (2017). Epidemiological Study of *Fusarium* Species Causing Invasive and Superficial Fusariosis in Japan. Med. Mycol. J..

[179] Viegas C., Dias R., Gomes A.Q., Meneses M., Sabino R., Viegas S. (2014). Aspergillus flavus Contamination in Two Portuguese Wastewater Treatment Plants. J. Toxicol. Environ. Health Part A.

